# Laparoscopic splenectomy for isolated splenic sarcoidosis: A case report

**DOI:** 10.1016/j.ijscr.2019.04.031

**Published:** 2019-04-19

**Authors:** Manabu Mikamori, Masahiro Tanemura, Kenta Furukawa, Takuro Saito, Masahisa Ohtsuka, Yozo Suzuki, Mitsunobu Imasato, Kentaro Kishi, Hiroki Akamatsu

**Affiliations:** Department of Surgery, Osaka Police Hospital, Kitayama-cho 10-31, Tennozi-ku, Osaka, 543-0035, Japan

**Keywords:** Sarcoidosis, Laparoscopy, Splenectomy, Case report

## Abstract

**Introduction:**

Isolated splenic sarcoidosis is difficult to diagnosis due to its rarity. Laparoscopic splenectomy has become the gold standard for therapeutic diagnosis in patients presenting with solid splenic lesions because needle biopsy can lead to bleeding and tract seeding.

**Presentation of case:**

A 59-year-old female was referred to our hospital due to abnormal accumulation in the spleen on abdominal ultrasonography. Enhanced computed tomography showed three heterogeneously enhanced nodules. Magnetic resonance imaging showed hypointense nodules on T2-weighted images. The initial diagnosis was a fibrous hamartoma or an inflammatory pseudotumor. At follow-up 4 months later, the splenic nodules had increased in size, and diagnostic laparoscopic splenectomy was performed without complications. Histopathologically, the splenic nodules contained noncaseating granulomas comprising epithelioid cells, multinucleated giant cells, and asteroid inclusion bodies. Postoperatively, examinations found no other organ involvement, and the final diagnosis was isolated splenic sarcoidosis. There was no evidence of recurrence at 2 years postoperatively, and systemic treatment was not required.

**Conclusion:**

Radiological imaging studies are insufficient for the differential diagnosis of splenic lesions in sarcoidosis from other diseases, whereas laparoscopic splenectomy is less invasive and useful as part of the diagnostic approach.

## Highlights

• Isolated splenic sarcoidosis is difficult to diagnose due to its rarity.
• Laparoscopic splenectomy has become the gold standard in patients presenting with solid splenic lesions.
• Laparoscopic splenectomy is less invasive and useful as part of the diagnostic approach.

## 1 Introduction

Sarcoidosis is a systemic disease of unknown origin characterized by the presence of noncaseating granulomatous lesions [1]. One of the extensively supported hypotheses states that sarcoidosis is an outcome of immune responses to various ubiquitous environmental triggers in patients with a genetic predisposition [2]. The presentation of sarcoidosis varies widely, affecting individuals of all racial and ethnic backgrounds, and can occur at any age. More than 90% of affected patients have pulmonary manifestations at diagnosis [3]. Extrapulmonary involvement usually occurs in the presence of lung involvement, whereas isolated extrapulmonary manifestations of sarcoidosis are rare, occurring in only 10% of the patients [4]. Specifically, isolated splenic sarcoidosis is difficult to diagnose due to its rarity. In patients presenting with solid splenic lesions, which are rare, differential diagnosis may include lymphoma, tumor metastasis, angiosarcoma, fibrous hamartoma, and inflammatory pseudotumor [5], in addition to splenic sarcoidosis. Biopsy may be indicated for definitive diagnosis. Needle biopsy can lead to bleeding and tract seeding; therefore, laparoscopic splenectomy has become the standard approach for therapeutic diagnosis. Herein, we report a patient with primary splenic sarcoidosis who was diagnosed and treated using laparoscopic splenectomy. The work in this case has been reported in line with the SCARE criteria [6].

## 2 Presentation of case

**Fig. 1:**
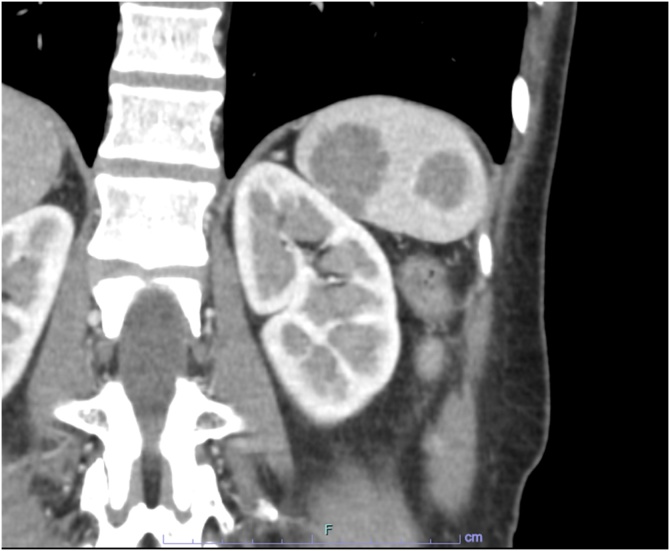
Abdominal enhanced computed tomography showing multiple hypoechoic nodules in the spleen.

**Fig. 2:**
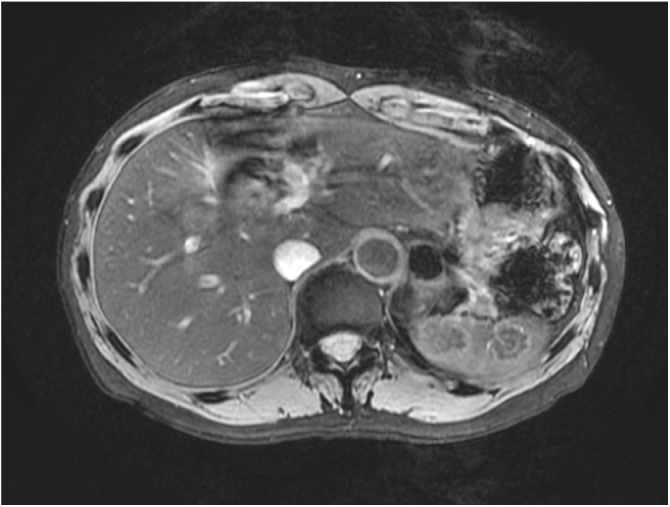
Magnetic resonance imaging of the abdomen showing hypointense nodules on T2-weighted images.

A 59-year-old female was referred to our hospital with abnormal nodules in the spleen on abdominal ultrasonography during a routine health screening. Her past medical history included an abscess in the fallopian tube and bilateral ovaries 7 years ago. Baseline laboratory tests, including complete blood count, serum levels of electrolytes (including calcium), renal and liver function tests, soluble interleukin-2 receptor (sIL-2R) level, and levels of the tumor markers carcinoembryonic antigen (CEA) and carbohydrate antigen 19-9 (CA19-9), were within normal limits. Abdominal ultrasonography revealed a normal-sized spleen with three small hypoechoic nodules of different dimensions. Enhanced computed tomography (CT) and magnetic resonance imaging (MRI) of the abdomen confirmed the presence of multiple lesions in the spleen. The three splenic nodules exhibited heterogeneous enhancement with a hypointense signal on T2-weighted images and measured 22, 17, and 9 mm in diameter (Fig. 1, Fig. 2). No other lesions were found in other organs.

The initial differential diagnosis included a fibrous hamartoma and an inflammatory pseudotumor, and a follow-up imaging was planned. An enhanced CT scan performed 4 months later revealed an increase in the diameters of all nodules to 27, 22, and 16 mm. Aberrant ^18^F-fluorodeoxyglucose (FDG) uptake with a maximum standardized uptake value (SUV_max_) of 2.89 was observed by positron emission tomography (PET)-CT, and the patient finally underwent diagnostic laparoscopic splenectomy.

The 4-trocar technique was used, and ligaments and vessels were dissected using a LigaSure^™^ (Valleylab, Boulder, CO, USA) or by clipping. After full mobilization of the viscus, the splenic artery was exposed until the peripheral parts, and the peripheral artery to the pancreas (pancreatic tail artery) was preserved. The spleen was removed through a small 3-cm opening in the umbilical region. There was no significant bleeding, and the total surgery time was 148 min. The patient recovered uneventfully and was discharged on postoperative day 5 with adequate oral intake, normal vital signs, and no complaints.

**Fig. 3:**
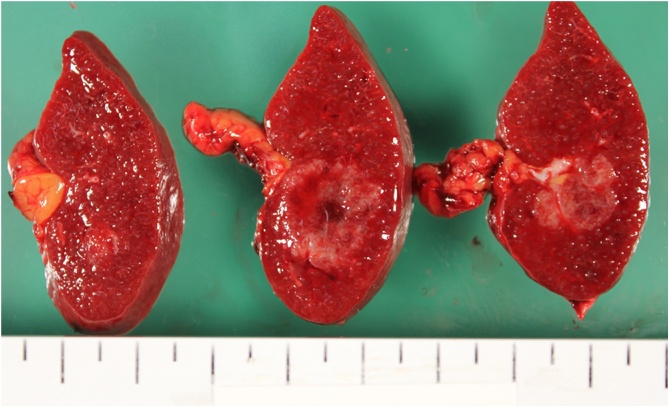
The resected specimen showing poorly demarcated white nodules.

**Fig. 4:**
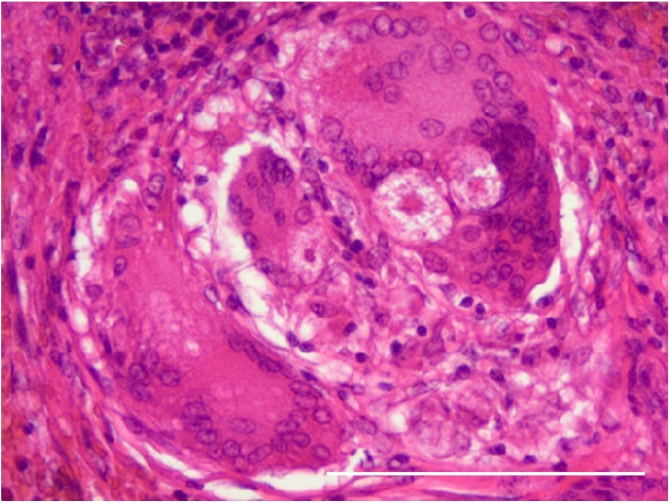
Histopathological examination showing noncaseating granulomas comprising multinucleated giant cells and asteroid inclusion bodies. Scale bar: 100 μm.

Gross examination revealed that the resected spleen contained poorly demarcated, white nodules (Fig. 3). Histopathological examination showed splenic parenchyma with discrete, round to oval noncaseating granulomas comprising epithelioid granulomas, multinucleated giant cells, and asteroid inclusion bodies (Fig. 4). Postoperatively, CT scan found that no other organs were involved, including the lung, heart, and eyes. The final diagnosis was isolated splenic sarcoidosis. The patient did not receive any systemic treatment. She had been following a steady favorable postoperative course without complications or disease recurrence at 2 years after surgical splenectomy.

## 3 Discussion

The diagnosis of sarcoidosis, relies on clinical and radiological imaging findings associated with the histology of epithelioid granulomas; however, granulomas are not pathognomonic for sarcoidosis. Sarcoidal granulomas can involve any organ, but clinical sarcoidosis manifests as intrathoracic lymph node enlargement, pulmonary involvement, skin or ocular signs and symptoms, or a combination of these findings in more than 90% of the patients. Conversely, splenic sarcoidosis in the absence of specific clinical or radiographic signs of pulmonary sarcoidosis is extremely rare, with few noteworthy reported cases [5,7,8]. Laboratory tests are not usually helpful for definite diagnosis. Radiological imaging findings of isolated splenic lesions are also nonspecific, and their differential diagnosis includes tumor metastasis, hemangioma, fibrous hamartoma, inflammatory pseudotumor, lymphoma, abscess, and angiosarcoma [9]. Therefore, histopathological diagnosis is necessary for the definitive diagnosis of isolated splenic lesions.

In the present case, abdominal ultrasonography revealed small hypoechoic nodules of different sizes, which were confirmed by CT, whereas MRI yielded no diagnostic advantage. The initial diagnosis was a benign tumor such as a fibrous hamartoma or an inflammatory pseudotumor. After 4 months of follow-up, diagnostic laparoscopic splenectomy was performed due to tumor size progression and aberrant uptake of 18F-FDG on PET-CT.

Laparoscopic splenectomy has been extensively employed as an effective approach for diagnosis and treatment of diseases and determination of malignancy involving the spleen [10]. The procedure is minimally invasive and provides significant benefits, including shorter hospital stay, better esthetics, and reduced blood loss and postoperative complications [5]. There are several clinical benefits of rapid recovery, including earlier initiation of chemotherapy. Splenectomy does not alter the course of disease but might be necessary for treating complications, and its indications include symptoms such as abdominal pain, hypersplenism, prophylaxis for splenic rupture, and exclusion of neoplasms [11]. Importantly, laparoscopic splenectomy has become the gold standard for surgical removal of the spleen.

The current case highlights the diagnostic approach for splenic lesions and the outcome of laparoscopic splenectomy as an effective and safe procedure for diagnosis and treatment. The present case was an extremely rare form of sarcoidosis. Isolated splenic involvement in sarcoidosis is a precursor for systemic disease. Although the current patient did not show recurrence of additional lesions of sarcoidosis at 2 years of follow-up, further monitoring is necessary to identify potential recurrence.

## Conflicts of interest

All authors declare that they have no conflicts of interest in relation to this study.

## Sources of funding

None of the authors has any conflicts of interest or any financial ties to disclose.

## Ethical approval

This study has been approved by the ethics committee in the Osaka Police Hospital. The ethical approval number is No. 912.

## Consent

Written informed consent was obtained from the patient for publication of this case report and accompanying images. A copy of the written consent is available for review by the Editor-in-Chief of this journal on request

## Author contribution

Conception and design of study: Manabu Mikamori, Masahiro Tanemura

Acquisition of data: Manabu Mikamori, Kenta Furukawa

Drafting the manuscript: Manabu Mikamori

Revising the manuscript critically for important intellectual content: Masahiro Tanemura, Kenta Furukawa, Takuro Saito, Masahisa Ohtsuka, Yozo Suzuki, Mitsunobu Imasato, Kentaro Kishi, Hiroki Akamatsu

## Registration of research studies

No registration of research studies.

## Guarantor

On behalf of all author, Manabu Mikamori is guarantor for this paper.

## Provenance and peer review

Not commissioned, externally peer-reviewed.
